# Cost-effectiveness analysis of metronomic capecitabine as adjuvant chemotherapy in locoregionally advanced nasopharyngeal carcinoma

**DOI:** 10.3389/fonc.2022.904372

**Published:** 2022-09-13

**Authors:** Longjiang She, Kun Tian, Jiaqi Han, Weihan Zuo, Zhu Wang, Ning Zhang

**Affiliations:** ^1^ Department of Radiation Oncology, First People’s Hospital of Foshan, Foshan, China; ^2^ Department of Urology, Institute of Urology, West China Hospital, Sichuan University, Chengdu, China; ^3^ Department of Head and Neck Oncology and Department of Radiation Oncology, Cancer Center and State Key Laboratory of Biotherapy, West China Hospital, Sichuan University, Chengdu, China; ^4^ Multi-omics Laboratory of Breast Diseases, West China Hospital, Sichuan University, Chengdu, China

**Keywords:** cost-effectiveness, metronomic chemotherapy, adjuvant chemotherapy, capecitabine, nasopharyngeal carcinoma

## Abstract

**Background:**

Adding metronomic capecitabine to concurrent chemoradiotherapy (CCRT) brings failure-free survival (FFS) benefits to patients with locoregionally advanced nasopharyngeal carcinoma (NPC). This study assesses the cost-effectiveness of metronomic capecitabine in locoregionally advanced NPC.

**Methods:**

We created a Markov model to calculate the expense and health outcomes of metronomic capecitabine compared to those observed in locoregionally advanced NPC. Related costs, like life-years (LYs), quality-adjusted life years (QALYs), and incremental cost-effective ratios (ICERs) were measured at a willingness-to-pay (WTP) threshold of $33,585 per QALY. A combination of different sensitivity analyses was used to test for model robustness. Additionally, a subgroup analysis was also performed.

**Results:**

In contrast to what is observed in the locoregionally advanced NPC, adding the metronomic adjuvant capecitabine yielded an additional 1.11 QALYs with an incremental cost of $10,741.59, which obtained an ICER of $9,669.99 per QALY. The result of one-way sensitive analysis indicated that the utility of FFS, progression disease (PD), and the cost of follow-up were the most significant factors. The probability of metronomic capecitabine being cost-effective was 97.1% at a WTP of $33,585 per QALY.

**Conclusion:**

Metronomic capecitabine as adjuvant chemotherapy is a cost-effective strategy for locoregionally advanced NPC patients.

## Introduction

Nasopharyngeal carcinoma (NPC) is a head and neck cancer that arises from the nasopharyngeal mucosal lining and is mostly observed in South China and Southeast Asia (1). Approximately 70% of patients with newly diagnosed NPC are classified as being locoregionally advanced (2). Platinum-based concurrent chemoradiotherapy (CCRT) with or without induction chemotherapy is the standard-of-care treatment for those patients (3). Although most patients will achieve clinical remission after standard therapy, approximately 30% of patients will either locoregionally relapse or develop distant metastatic disease (2, 4–7).

Recently, metronomic capecitabine as adjuvant therapy was useful in locoregionally advanced NPC based on the data of NCT02958111 (8). In this trial, patients with locoregionally advanced NPC who had no locoregionally or distant metastatic disease after standard-of-care treatment were enrolled (8). Eligible patients will be given metronomic capecitabine or observation. Patients in the metronomic capecitabine group had a higher failure-free survival (FFS) (85.3% vs 75.7%, p = 0.0023) at three years than those under observation. Although the incidence rate was higher in the metronomic capecitabine group of grade 3 adverse events (AEs) compared with the observation group (17% vs 6%), they had a similar health-related quality of life (HRQOL) until disease progression (8). Metronomic adjuvant capecitabine is an alternative for high-risk locoregionally advanced NPC patients recommended by the 2021 Chinese Society of Clinical Oncology (CSCO) guideline, with compelling clinical benefits and no HRQOL detriment.

While metronomic capecitabine as adjuvant therapy could be beneficial for patients with locoregionally advanced NPC, it is still unclear whether metronomic capecitabine is cost-effective compared with observation. We estimated the cost-effectiveness of metronomic capecitabine as adjuvant therapy in locoregionally advanced NPC. Since the clinical trial was conducted in China, we analyzed the results from the perspective of China as well.

## Materials and methods

### Model structure

The Markov model was developed to measure the cost-effectiveness of the metronomic adjuvant capecitabine in locoregionally advanced NPC by using TreeAge Pro 2019 (TreeAge Software Inc., Williamstown, MA). According to the treatment cycle in the trial, we had set three weeks as the Markov cycle length. We applied a commonly used discount rate per year of 5% in China (9). The main outcomes included total costs, life years (LYs), quality-adjusted life years (QALYs), and incremental cost-effectiveness ratios (ICERs). They were estimated across metronomic capecitabine and observation treatment groups, respectively.

Three states, FFS, progression disease (PD), and death, were used to simulate the development process of a locoregionally advanced NPC (**Supplementary Figure 1**). After standard-of-care treatment, eligible patients were treated with metronomic capecitabine or observation in the FFS state until progression or unacceptable toxic effects. After progression, both metronomic capecitabine and observation groups could receive subsequent therapy according to the data of NCT02958111.

### Model survival and transitions estimates

Based on the data of FFS and overall survival (OS) in the NCT02958111 trial, we assessed the transition probabilities between each health state through the following steps: Firstly, we used the software GetData Graph Digitizer (version 2.25; http://www.getdata-graph-digitizer.com/index.php) to collect the data points from the OS Kaplan–Meier curves of metronomic capecitabine and the observation group (10), which were assigned to fit parametric survival models. There are a series of parametric survival models that are commonly used in cost-effectiveness analysis, including the Log-logistic, Exponential, Weibull, Lognormal, and Gompertz distributions (11). However, we applied Log-logistic distribution because it had the lowest Akaike’s information criterion (AIC) and Bayesian information criterion (BIC) values. This implied that a Log-logistic distribution could better fit the survival curve than the other four distributions (**Supplementary Figures 2, 3**, and **Table 2**). Next, the R software package (http://www.r-project.org/) was used to generate the shape parameter (γ) and the scale parameter (λ), which were estimated from this fit.

### Utility estimates

The clinical trial used the European Organization for Research and Treatment of Cancer (EORTC) Quality of Life Questionnaire-Core 30 (QLQ-C30) version 3.0, a paper-based questionnaire to measure HRQOL (8). The results showed no substantial difference in baseline and HRQOL detriment while performing adjuvant therapy with metronomic capecitabine (8). Thus, similar utilities in both metronomic capecitabine and observation groups were used. Consistent with earlier literature, utilities of 0.76 and 0.57 were applied to patients in FFS and PD states, respectively (12).

### Cost Inputs

Direct medical costs such as drugs, radiotherapy, hospitalization, follow-up, laboratory tests, management of AEs, and subsequent therapy were included in the model. Standard-of-care treatment was recommended for eligible patients, which included CCRT with or without induction chemotherapy (two or three cycles). There were three induction chemotherapy regimens: gemcitabine and cisplatin, docetaxel and cisplatin, and docetaxel, cisplatin, and fluorouracil. These patients received metronomic capecitabine or observation as adjuvant therapy. For the metronomic capecitabine group, patients received oral capecitabine 650 mg/m^2^ twice daily for 1 year (the dose was reduced by 25% in 14% of the patients and by 50% in 4% of the patients). Patients also received subsequent therapy after disease progression based on the trial of NCT02958111 (8).

We have applied a standard body surface area of 1.72 m^2^ derived from similar research reported in China (13). We also took grade 3 or higher AEs with a frequency of greater than 1% into consideration. All costs were derived from earlier literature and the First People’s Hospital of Foshan (9, 14–16). All costs were adjusted to United States dollars (USD) (1 USD = 6.47 CHY; February 2021). The details are listed in **Tables 1**, **2**.

**Table 1:** Key clinical data in trial of NCT02958111.

Variable	Baseline value (Range)	Reference	Distribution
**FFS survival model**			–
Metronomic capecitabine	Shape = 1.2627183, Scale = 0.0011293	(8)	–
Observation	Shape = 1.0422333, Scale = 0.0060393	(8)	–
**OS survival model**			–
Metronomic capecitabine	Shape = 1.64, Scale = 0.00009264	(8)	–
Observation	Shape = 1.4009241, Scale = 0.0005708	(8)	–
**Risk for main adverse events in metronomic capecitabine**			–
Leukopenia	0.03	(8)	Beta
Neutropenia	0.03	(8)	Beta
Hand-foot syndrome	0.09	(8)	Beta
Nausea	0.01	(8)	Beta
Sensory neuropathy	0.01	(8)	Beta
**Risk for main adverse events in observation**			
Leukopenia	0.03	(8)	Beta
Neutropenia	0.03	(8)	Beta
Anemia	0.01	(8)	Beta
Sensory neuropathy	0.01	(8)	Beta
**Proportion of induction chemotherapy in metronomic capecitabine**			
Yes	0.77	(8)	–
No	0.23	(8)	–
**Proportion of induction chemotherapy in observation**			
Yes	0.78	(8)	–
No	0.22	(8)	–
**Proportion of different induction chemotherapy regimes in metronomic capecitabine**			
TP	0.72	(8)	–
TPF	0.22	(8)	–
GP	0.06	(8)	–
**Proportion of different induction chemotherapy regimes in observation**			
TP	0.76	(8)	–
TPF	0.18	(8)	–
GP	0.06	(8)	–
**Proportion of different cycles of induction chemotherapy in metronomic capecitabine**			
2	0.28	(8)	–
3	0.72	(8)	–
**Proportion of different cycles of induction chemotherapy in observation**			
2	0.29	(8)	–
3	0.71	(8)	–

FFS, failure-free survival; GP, gemcitabine and cisplatin; OS, overall survival; TP, docetaxel and cisplatin; TPF, docetaxel cisplatin and fluorouracil.

**Table 2:** Cost estimates and utilities.

Variable	Baseline value (Range)	Reference	Distribution (parameters)
**Body surface area, m^2^**	1.72	(13)	–
**Drug cost, $/cycle**			–
Capecitabine	41.07 (32.86–49.28)	Local charge	Gamma
TP	42.83 (34.26–51.39)	Local charge	Gamma
TPF	266.46 (213.17–319.76)	Local charge	Gamma
GP	35.29 (28.24–42.35)	Local charge	Gamma
Concurrent cisplatin	17.54 (14.04–21.05)	Local charge	Gamma
**Radiotherapy**	9,633.84 (7,707.07–11,560.61)	Local charge	Gamma
**Preparation of radiotherapy**	681.52 (545.22–817.83)	Local charge	Gamma
**Subsequent therapy in capecitabine**	152.66 (122.13–183.19)	Local charge	Gamma
**Subsequent therapy in observation**	244.89 (179.91–269.87)	Local charge	Gamma
**Expenditures on main adverse events, $**			–
Anemia	508.2 (406.56–609.84)	(9)	Gamma
Leukopenia	406.37 (325.10–487.64)	(14)	Gamma
Neutropenia	406.37 (325.10–487.64)	(14)	Gamma
Nausea	44.3 (35.44–53.16)	(16)	Gamma
Hand-foot syndrome	773.64 (618.11–927.17)	Local charge	Gamma
Sensory neuropathy	29.78 (23.82–35.74)	Local charge	Gamma
**Hospitalization $/per cycle**	126.78 (101.43–152.14)	Local charge	Gamma
**Laboratory $/per cycle**	113.39 (90.71–136.07)	Local charge	Gamma
**Follow-up test $/per time**	550.59 (440.47–660.71)	Local charge	Gamma
**Discount rate**	0.05	(9)	–
**Utility**			–
Utility FFS	0.76 (0.61–0.91)	(12)	Beta
Utility PD	0.57 (0.46–0.68)	(12)	Beta
Death	0	(12)	Beta

FFS, Failure-free survival; GP, gemcitabine and cisplatin; PD, progressive disease; TP, docetaxel and cisplatin; TPF, docetaxel cisplatin and fluorouracil.

### Sensitivity analysis

Both one-way and probabilistic sensitivity analyses were performed to test and check the robustness of the model. We applied one-way sensitivity analysis to test the effect of each input parameter, and the probabilistic sensitivity analysis to simultaneously assess input parameters which were derived from statistical distributions by 10,000 resampling. Besides, we developed structural sensitive analysis to estimate the cost-effectiveness of metronomic capecitabine for three years. Subgroup analysis was also conducted to find the advantaged population.

## Results

### Base case results

In comparison to the observation group, metronomic capecitabine produced an additional 1.65 LYs. Metronomic capecitabine generated 8.18 QALYs at a cost of $70,375.42, while the observation group provided 7.07 QALYs at $59,633.84. Hence, an ICER of $6,499.18 per LY and $9,668.99 per QALY was noted for the metronomic capecitabine group over the observation group (**Table 3**).

**Table 3:** Baseline results.

Strategies and Scenarios	Total cost, $	LYs	QALYs	ICER per LY, $/LY	ICER per QALY, $/QALY
Metronomic capecitabine	70,375.42	11.78	8.18	6,499.18	9,668.99
Observation	59,633.84	10.13	7.07	–	–

ICER, incremental cost-effectiveness ratio; LY, life-year; QALY, quality-adjusted life-year.

### Sensitivity analysis

**Figure 1:**
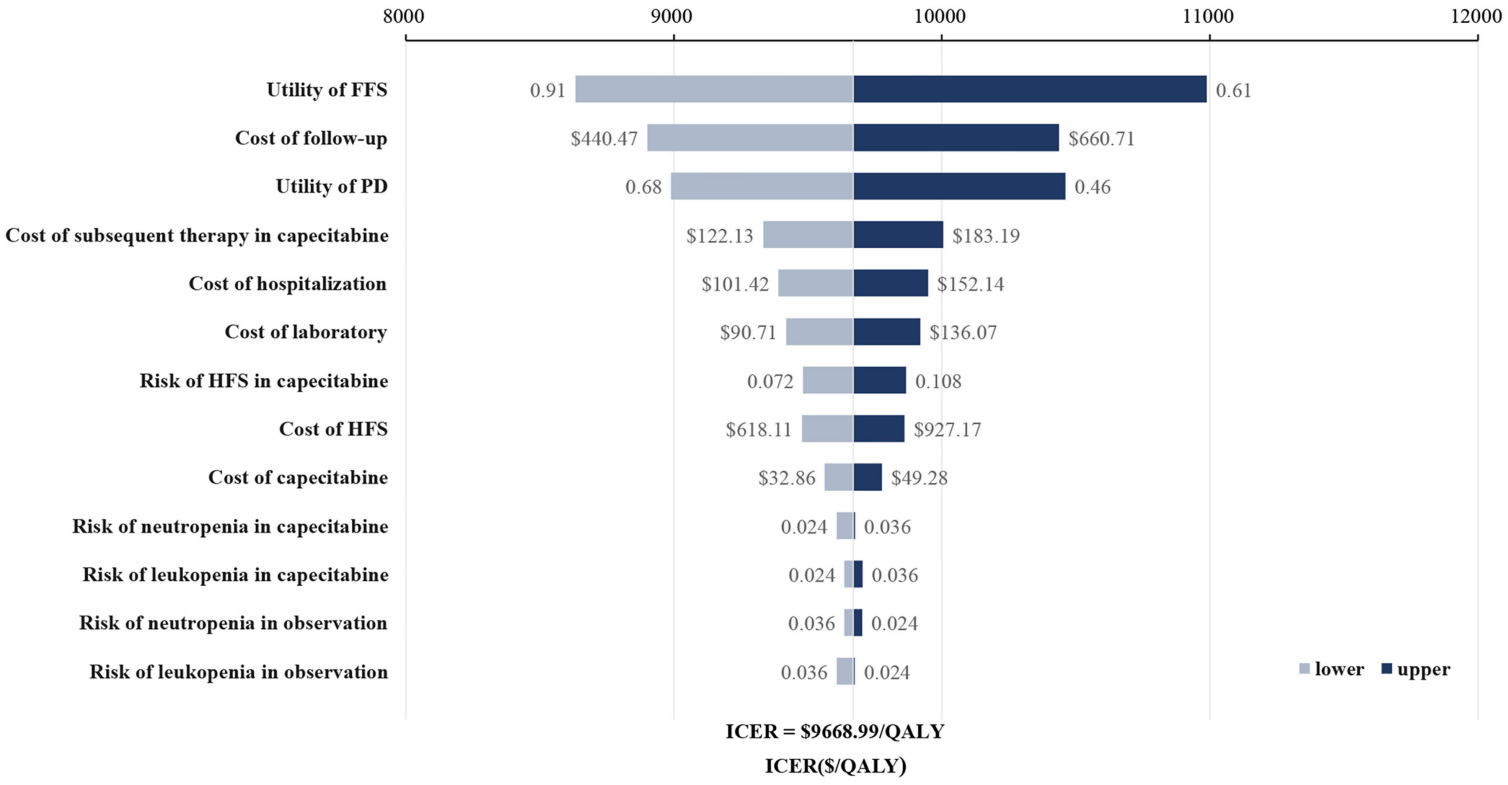
Tornado diagram for one-way sensitivity analysis. FFS, failure-free survival; HFS, hand-foot syndrome; PD, progressive disease.

**Figure 2:**
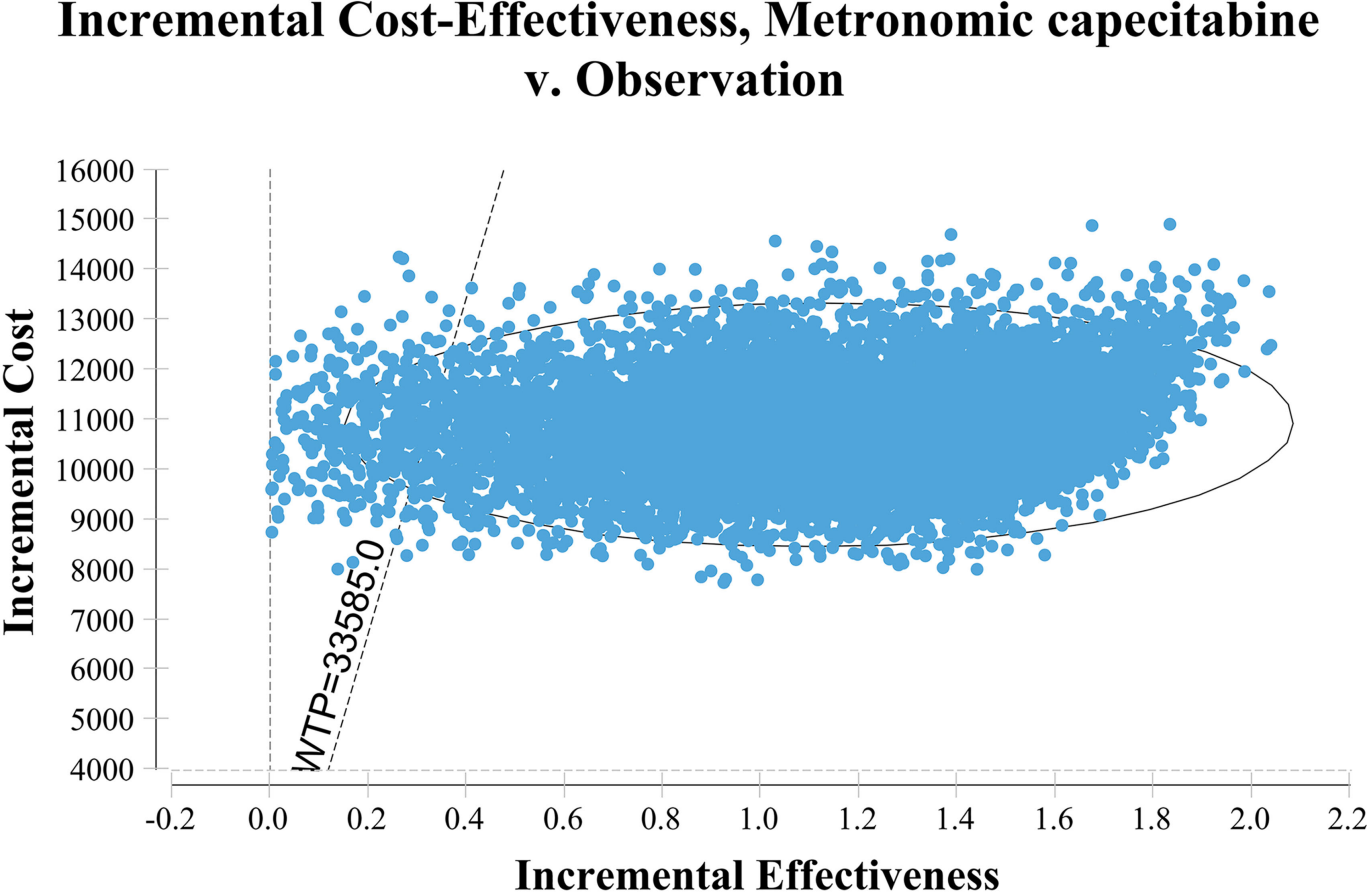
The results of probabilistic sensitivity analysis.

**Figure 3:**
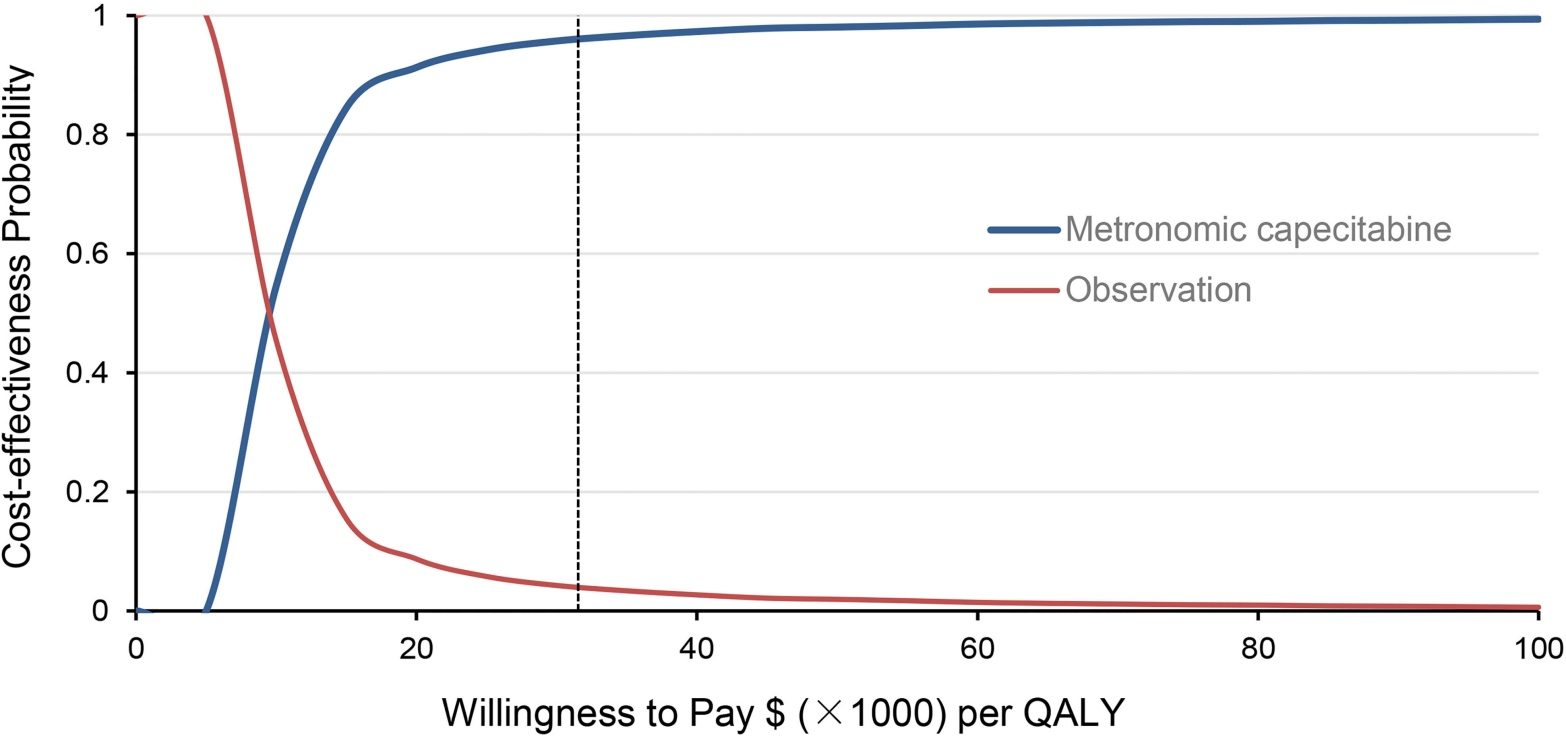
Acceptability curves of cost-effectiveness probability for metronomic capecitabine and observation treatment at different willingness-to-pay (WTP) values in locoregionally advanced nasopharyngeal carcinoma (NPC).

**Figure 1** shows the one-way sensitivity analysis through a tornado diagram. The utility of FFS, the cost of follow-up, and the utility of PD were found to have a significant influence on the economic outcomes. Subsequently, probabilistic sensitivity analysis indicated that the probability of metronomic capecitabine being cost-effective was 97.1% compared to the observation at a willing-to-pay (WTP) threshold of $33,585 (**Figures 2**, **3**) (17).

In structural sensitivity analysis, the ICER of extended three years of metronomic capecitabine treatment was $12,214.37 per QALY, lower than the value of WTP (**Table 4**). Similar results were observed in all subgroups by the subgroup analysis (**Supplementary Table 1**).

**Table 4:** Results of structural sensitivity analysis.

Strategies and Scenarios	Total cost, $	LYs	QALYs	ICER per LY, $/LY	ICER per QALY, $/QALY
Capecitabine for three years	–	–	–	–	–
Metronomic capecitabine	73,895.58	11.78	8.18	8,210.10	12,214.37
Observation	60,326.25	10.13	7.07	–	–

ICER, incremental cost-effectiveness ratio; LY, life-year; QALY, quality-adjusted life-year.

## Discussion

Historically, adjuvant chemotherapy has had limited importance in NPC (18). The new trial of NCT02958111 revealed that metronomic adjuvant capecitabine after standard-of-care treatment could improve FFS in locoregionally advanced NPC. However, it is necessary to assess whether a new anti-cancer treatment is cost-effective before it is recognized by patients and oncologists, especially in developing countries.

Because of the high incidence of NPC in China, cost-effectiveness analyses of NPC were performed in China. Two cost-effectiveness analyses compared gemcitabine and cisplatin with fluorouracil and cisplatin. They indicated that gemcitabine and cisplatin were the most cost-effective regimens for patients with metastatic NPC in China (13, 19). A cost-effectiveness analysis of different concurrent chemotherapy regimens demonstrated that a comparison of nedaplatin with cisplatin was not a cost-effective strategy (20). For induction chemotherapy, research conducted by Wu demonstrated that the ICER of gemcitabine plus cisplatin was $2,804.44 per QALY compared with docetaxel, cisplatin, and fluorouracil (21). Another study developed by Yang showed that gemcitabine plus cisplatin produced an additional 0.42 QALYs with an incremental cost of $3,821.99. This yielded an ICER of $9,099.98 per QALY for the gemcitabine plus cisplatin regime over the docetaxel, cisplatin, and fluorouracil regimes (12). Both analyses revealed that gemcitabine plus cisplatin was a cost-effective induction chemotherapy choice for locoregionally advanced NPC in China (12, 21).

This is the first study to estimate the cost-effectiveness of metronomic capecitabine compared with observation as adjuvant therapy in patients with locoregionally advanced NPC. The ICER value was found to be significantly lower than the value of WTP and even lower than the one-time per capita gross domestic product (GDP). The tornado diagram of the one-way sensitive analysis showed that metronomic capecitabine was still cost-effective no matter how each input parameter changed within the plausible range. The probabilistic sensitivity analysis suggested that metronomic capecitabine was 97.1% cost-effective. Given the different characteristics in the intention-to-treat population, subgroup analysis was performed to identify the subgroups with a significant benefit in cost-effectiveness. The results showed that the ICER of metronomic capecitabine versus observation was lower than the value of WTP in all patient subgroups, which indicated that metronomic capecitabine was cost-effective in all patient subgroups shown in **Supplementary Table 1**. For patients with locoregionally advanced NPC, treatment with metronomic capecitabine after standard-of-care treatment could generate survival benefits and is a cost-effective choice. These may inform clinicians, regulators, or patients when choosing treatment regimes.

Due to its spatial and geographical location, there is an imbalance in the economic development across the provinces of China. Therefore, the effect of economic development in each province should also be considered. According to the National Bureau of Statistics (2020), Gansu had the lowest GDP per capita ($5,571.13), while Beijing had the highest GDP per capita ($25,485.97) (22). The corresponding WTP range was $16,713.39 to $76,457.91 per QALY. Hence, based on the base case results, metronomic capecitabine was considered cost-effective even in Gansu. This was also confirmed by the sensitivity analysis. Thus, metronomic capecitabine could be used in most places in China at acceptable costs. Our results suggest analyzing patients in regions where the use of metronomic capecitabine can be cost-effective.

Furthermore, the purpose of the NCT02958111 trial was to estimate the effect of metronomic capecitabine treatment for one year. One study suggested that the first three years after chemoradiotherapy are the highest incidence time of treatment failure (23). In another study associated with metastatic NPC, the median follow-up duration was 33.8 months, and the results showed that capecitabine maintenance could increase the chances of survival (24). However, it is still unclear whether the perfect duration of metronomic treatment is one year or not. Thus, we used the structural sensitivity analysis and extended the metronomic capecitabine treatment time by three years based on the published studies (23, 24). The ICER of metronomic capecitabine versus observation was $12,214.37 per QALY. Hence, metronomic capecitabine was still found to be a cost-effective treatment at a WTP value of $33,585 per QALY (**Table 4**). However, note that the survival benefits will be different with the extended treatment time. The result can differ from the actual situation since we have extended the treatment time only for similar patients. However, the ICER of metronomic capecitabine versus observation was still much lower than the value of WTP. Perhaps this information could be beneficial to the researchers of follow-up clinical trials.

We have considered factors pertinent to the results. However, we still acknowledge that there are still some limitations to our research. First, we established our model to project long-term survival results beyond the follow-up time of the trial, which may differ in the case of a real-world scenario. However, many studies based on cost-effectiveness have used the same method to simulate survival data (9, 25–28). This may underestimate or overestimate the clinical benefits of metronomic capecitabine. Second, many of the costs used in our analysis were derived from a single institution. Although this institution can represent the cost level of most hospitals in China for which we have set a reasonable range, the actual results may differ. Third, except for FFS, we assume that other characters are similar in the subgroup. Since it is an exploratory analysis, the results of the subgroup analysis should be interpreted with caution. Fourth, the NCT02958111 was a multicenter clinical trial created for the Chinese population, and the current cost-effectiveness analysis was also developed in China. Although a series of sensitivity analyses were conducted to verify the robustness of the model, we should be cautious in interpreting the conclusions in the case of other countries or regions.

In conclusion, the study suggested that metronomic capecitabine as adjuvant chemotherapy following standard-of-care treatment was more cost-effective than observation in patients with locoregionally advanced NPC in China.

## Data availability statement

The original contributions presented in the study are included in the article/**Supplementary Material**. Further inquiries can be directed to the corresponding author.

## Author contributions

Conceived and designed the experiments: LS, KT, and NZ. Performed the experiments: LS and JH. Analyzed the data: LS, WZ, and JH. Contributed reagents/materials/analysis tools: LS, ZW, and KT. Wrote the manuscript: LS, JH, and NZ. All authors read and approved the final manuscript.

## Conflict of interest

The authors declare that the research was conducted in the absence of any commercial or financial relationships that could be construed as a potential conflict of interest.

## Publisher’s note

All claims expressed in this article are solely those of the authors and do not necessarily represent those of their affiliated organizations, or those of the publisher, the editors and the reviewers. Any product that may be evaluated in this article, or claim that may be made by its manufacturer, is not guaranteed or endorsed by the publisher.

## Supplementary material

Supplementary Figure 1 Markov model states. The progress states of locoregionally advanced nasopharyngeal carcinoma (NPC).
