# Supplementary material for: Cost-effectiveness analysis of metronomic capecitabine as adjuvant chemotherapy in locoregionally advanced nasopharyngeal carcinoma
Source: Front Oncol. 2022 Sep 13;12:904372. doi: 10.3389/fonc.2022.904372 (PMC9513587; doi:10.3389/fonc.2022.904372)
Supplement: Supplementary file 1 [file Table_1.docx]

**Supporting Table 1. Results of subgroup analyses.**

| **Subgroup** | **FFS HR (95% CI)** | **ICER per QALY (95% CI)** | **Cost-effectiveness probability at WTP $33585/QALY** |
| --- | --- | --- | --- |
| **Sex** |  |  |  |
| Male | 0.48 (0.29-0.80) | 11887.59 (10702.80-12990.44) | 94.0% |
| Female | 0.55 (0.21-1.48) | 12194.99 (9938.53-14076.52) | 92.8% |
| **Age** |  |  |  |
| <46 years | 0.45 (0.22-0.90) | 11739.26 (10047.62-13221.59) | 94.2% |
| ≥46 years | 0.54 (0.30-0.99) | 12154.04 (10783.58-13401.05) | 93.0% |
| **ECOG** |  |  |  |
| 0 | 0.45 (0.25-0.81) | 11739.26 (10349.66-13015.29) | 94.2% |
| 1 | 0.58 (0.29-1.18) | 12312.61 (10702.80-13712.71) | 92.8% |
| **Tumor category** |  |  |  |
| T1-2 | 0.42 (0.11-1.61) | 11579.14 (8510.04-14200.32) | 94.6% |
| T3 | 0.54 (0.28-1.07) | 12154.04 (10619.20-13542.10) | 93.0% |
| T4 | 0.46 (0.23-0.92) | 11789.94 (10152.32-13263.59) | 94.1% |
| **Node category** |  |  |  |
| N1 | 0.66 (0.21-2.10) | 12592.49 (9938.53-14549.75) | 91.1% |
| N2 | 0.49 (0.27-0.90) | 11934.66 (10532.60-13221.60) | 93.9% |
| N3 | 0.44 (0.19-1.03) | 11687.27 (9705.81-13473.55) | 94.4% |
| **Disease stage** |  |  |  |
| III | 0.59 (0.29-1.20) | 12350.16 (10702.80-13741.22) | 92.6% |
| IVA | 0.45 (0.25-0.81) | 11739.26 (10349.66-13015.29) | 94.2% |
| **Induction chemotherapy regimen** |  |  |  |
| TP | 0.52 (0.28-0.97) | 12069.32 (10619.20-13363.21) | 93.2% |
| TPF | 0.36 (0.13-1.00) | 11216.87 (8859.00-13419.56) | 96.5% |
| GP | 0.50 (0.04-5.50) | 11980.60 (6822.27-15318.25) | 93.7% |
| **Number of cycles of induction chemotherapy** |  |  |  |
| 2 | 0.40 (0.14-1.12) | 11465.08 (9018.97-13622.71) | 94.9% |
| 3 | 0.52 (0.29-0.96) | 12069.32 (10702.80-13343.88) | 93.2% |

Abbreviation: CI, confidence interval; ECOG, Eastern Cooperative Oncology Group; FFS HR, failure-free survival hazard ratio; GP, gemcitabine and cisplatin; ICER, incremental cost-effectiveness ratio; LY, life-year; QALY, quality-adjusted life-year; TP, docetaxel and cisplatin; TPF, docetaxel, cisplatin and fluorouracil; WTP, Willingness-to-pay.
