# Supplementary material for: Cost-effectiveness analysis of metronomic capecitabine as adjuvant chemotherapy in locoregionally advanced nasopharyngeal carcinoma
Source: Front Oncol. 2022 Sep 13;12:904372. doi: 10.3389/fonc.2022.904372 (PMC9513587; doi:10.3389/fonc.2022.904372)
Supplement: Supplementary file 2 [file Table_2.docx]

|  | **Exponential** | **Weibull** | **Log-logistic** | **Lognormal** | **Gompertz** |
| --- | --- | --- | --- | --- | --- |
| **Metronomic capecitabine VS Observation** | | | | | |
| **Metronomic capecitabine OS curve** | | | | | |
| AIC | -425.8266 | -481.9824 | -494.3912 | -483.5835 | -466.1942 |
| BIC | -421.4473 | -475.4134 | -487.8223 | -477.0146 | -459.6252 |
| **Observation OS curve** | | | | | |
| AIC | -367.4345 | -403.0498 | -426.8659 | -407.6831 | -415.6498 |
| BIC | -363.0552 | -396.4808 | -420.2970 | -401.1141 | -409.0808 |
| **Metronomic capecitabine FFS curve** | | | | | |
| AIC | -428.7347 | -469.0935 | -490.2564 | -475.4038 | -465.8136 |
| BIC | -424.3554 | -462.5246 | -483.6875 | -468.8348 | -459.2447 |
| **Observation FFS curve** | | | | | |
| AIC | -350.2062 | -353.2517 | -388.7312 | -366.0288 | -348.4581 |
| BIC | -345.8269 | -346.6827 | -382.1622 | -359.4598 | -341.8891 |

**Supporting Table 2.** Summary of statistical goodness-of-fit of K-M curve in NCT02958111 trial.

Abbreviation: AIC, Akaike’s information criterion; BIC, Bayesian information criterion; FFS, failure-free survival; OS, overall survival.
