# Supplementary figures and images for: Cost-effectiveness analysis of metronomic capecitabine as adjuvant chemotherapy in locoregionally advanced nasopharyngeal carcinoma
Source: Front Oncol. 2022 Sep 13;12:904372. doi: 10.3389/fonc.2022.904372 (PMC9513587; doi:10.3389/fonc.2022.904372)

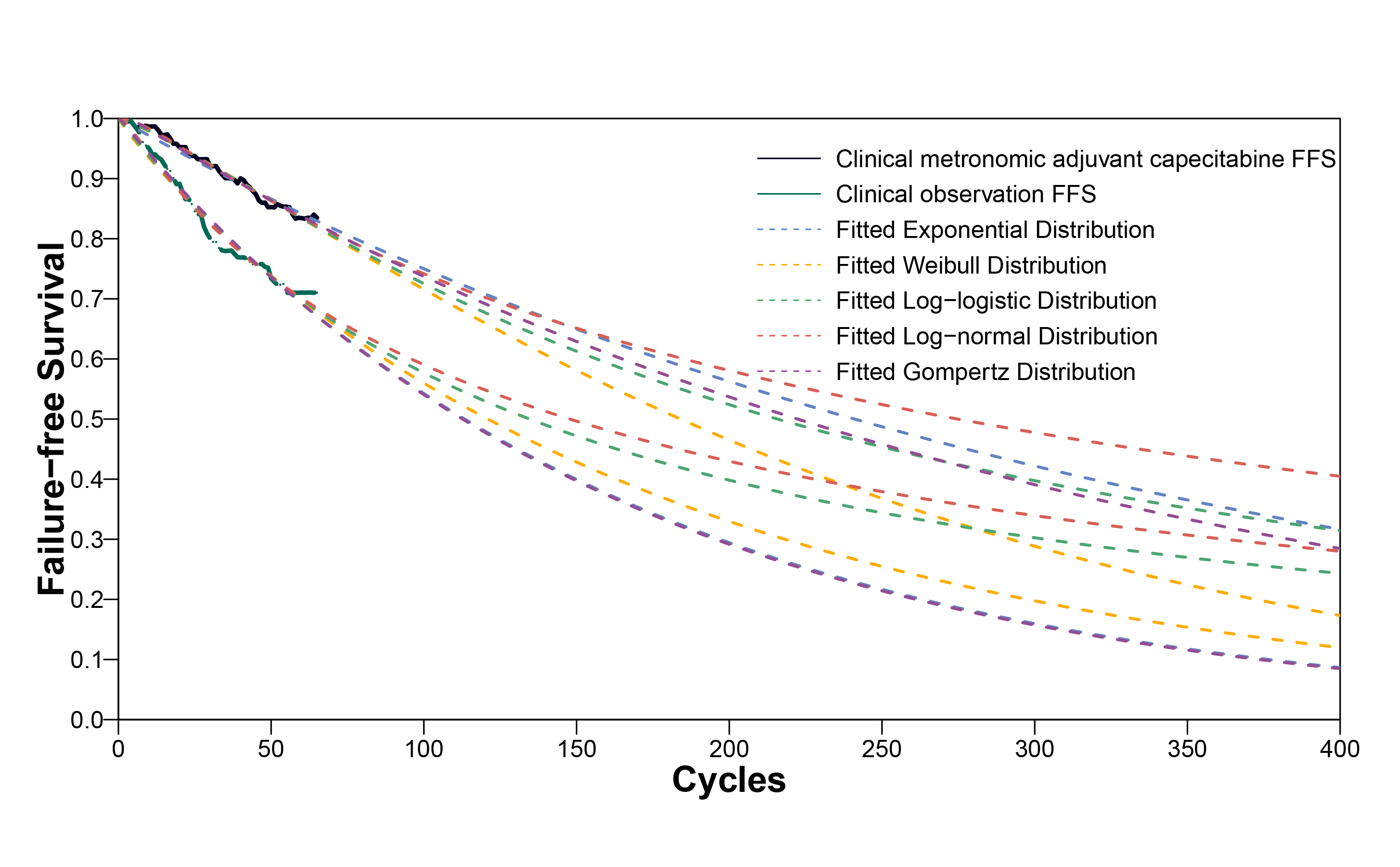

Supplement: Supplementary file 3 [file Image_2.jpeg]

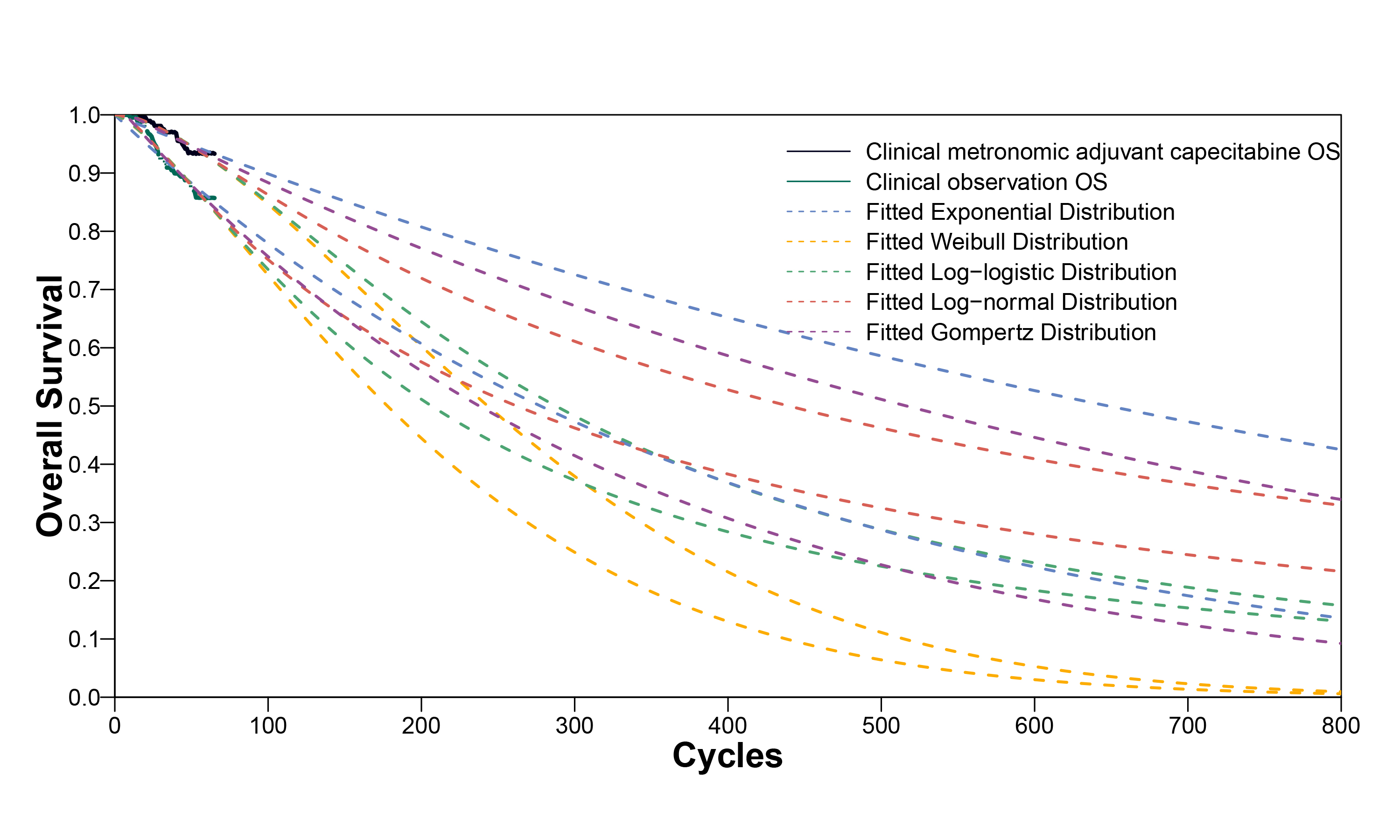

Supplement: Supplementary file 4 [file Image_3.jpeg]

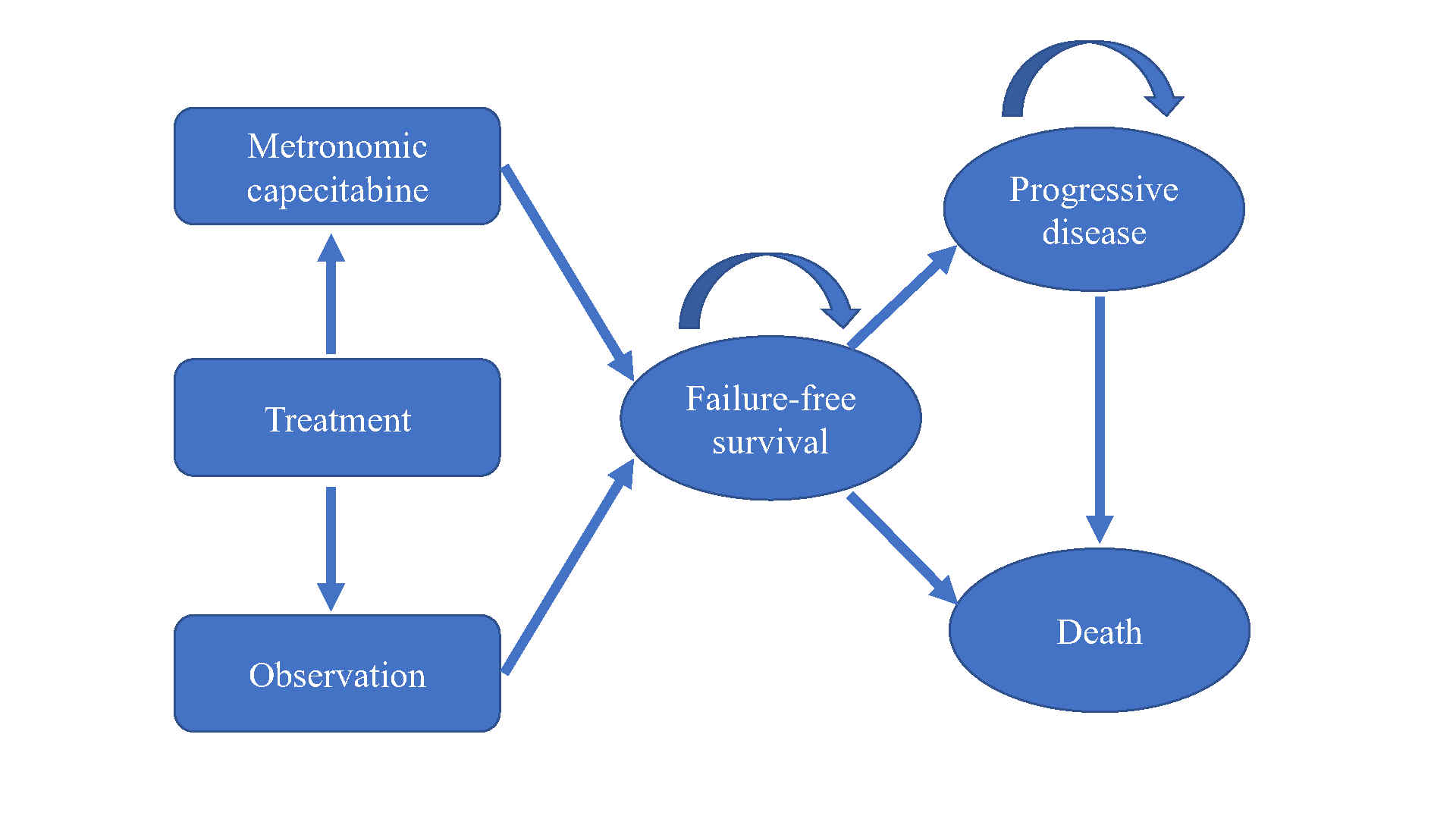

Supplement: Supplementary Figure 1 — Markov model states. The progress states of locoregionally advanced nasopharyngeal carcinoma (NPC). [file Image_1.jpeg]
